# Quantitative study on dose distribution of Freiburg flap for keloid high‐dose‐rate brachytherapy based on MatriXX

**DOI:** 10.1002/acm2.14118

**Published:** 2023-08-18

**Authors:** Jie Ni, Guanghui Gan, Xiaoting Xu

**Affiliations:** ^1^ Radiation Therapy Center The First Affiliated Hospital of Soochow University Suzhou Jiangsu Province China

**Keywords:** dose verification, HDR brachytherapy, MatriXX

## Abstract

**Purpose:**

To quantify the dose distribution effect of insufficient scattering conditions in keloid HDR brachytherapy with Freiburg fFlap (FF) applicator.

**Materials and Methods:**

A phantom composed of FF applicator, MatriXX and solid water slices was designed and scanned for treatment planning. Bolus with different thicknesses were covered to offer different scatter conditions. Planar dose distributions were measured by MatriXX. The maximum value (Max), average value (Avg) and γ passing rate (3 mm/3%) were evaluated by the software MyQA Platform.

**Results:**

The maximum and average doses measured by MatriXX were lower than the calculated values. The difference increased as field size decreased. The Max value, found at 0.86 cm level in the two tube case, was ‐20.0%, and the avg value was ‐11.9%. All the γ values were less than 95%. This difference gradually decreased with increasing bolus thickness and the γ values were significantly improved.

**Conclusion:**

MatriXX could be used for dose verification of HDR brachytherapy with an FF applicator. When the FF applicator was applied for keloid, insufficient scattering conditions would cause an insufficient target dose. This difference could be reduced by covering the bolus with different thicknesses on the applicator. The smaller the field, the thicker the bolus required.

## 1 INTRODUCTION

High‐dose‐rate brachytherapy(HDR‐BT)is a well‐recognized modality for keloid treatment after surgery in the clinic. A primary characteristic of HDR‐BT is the rapid and sharp dose fall‐off, resulting in more focus on the target and less exposure to surrounding skin.^1^, ^2^ The cosmetic outcome is proven to be satisfactory. The Freiburg flap (FF) applicator, which is a flexible mesh‐style surface module formed by 1 cm diameter spheres with catheters tunneled through, is widely used.^3^ Dose distribution according to different shapes varies for different patients. However, patient‐specific quality assurance is still lacking. End‐to‐end dose verification is necessary but not commonly used.^4^ The dosimetric algorithms applied in the brachytherapy treatment planning system (TPS) are mostly based on formulas in the AAPM TG43 report,^5^, ^6^ assuming that there is infinite uniform water around the radiation source, which provides sufficient accuracy in most cases.^7^ But for superficial skin tumors, the FF applicator placed on the skin surface cannot provide sufficient scattering conditions.^8^ Several studies have been carried out to evaluate the dosimetric effect of incomplete scattering conditions.^9^, ^10^, ^11^, ^12^, ^13^ Jayakody reviewed the dosimetry procedure to verify the dose in HDR brachytherapy.^14^ The TG‐186 report proposed a model‐based dose calculation algorithm^15^ and several commercial TPS have updated their program.^16^, ^17^ However, they were found unsuitable for clinical use.^17^, ^18^ Dose verifications in most of previous studies are completed by Monte Carlo calculation or film measurement, which is not always clinically available. Only a few studies are operated by MatriXX (IBA Dosimetry, Schwarzenbruck, Germany), a 2D ionization chamber array widely used in teletherapy. Manikandan et al. perform the mean dwell positional accuracy measurement with MatriXX.^19^ Jozef et al. conduct a study with line catchers to determine the suitability of MatriXX in measuring dose distribution. The results are within ± 10% with a 3 mm/3% γ index.^20^ This paper attempts to offer a feasible method for dose verification with FF applicator, and to quantitatively evaluate the delivered dose effect of insufficient scatter condition with FF applicator by MatriXX.

## 2 METHODS

### 2.1 Experimental setup and CT image acquisition

**FIGURE 1:**
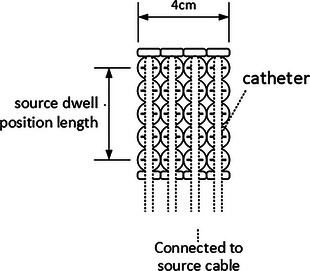
The FF applicator of 4 cm × 5 cm with a 4 cm × 5 cm field after regular cutting.

In the present study, the experimental phantom was constructed with the FF applicator set, MatriXX and 10 cm solid water equivalent slab (Figure 1), providing full back‐scattering conditions for MatriXX. The MatriXX Evolution consists of 1020 vented, plane‐parallel cylindrical ionization chambers with a maximum 24.4 × 24.4 cm^2^ field. The chamber size is 4.5 mm in diameter and 5 mm in height, center‐to‐center distance is 0.76 cm, and active volume is 0.08 cm^3^. Five sets of FF applicator were applied to simulate different target sizes: (A) 2 cm × 2 cm; (B) 4 cm × 4 cm; (C) 6 cm × 6 cm; (D) 8 cm × 8 cm; (E) 24 cm × 28 cm, respectively. For E target, a full‐size applicator set was utilized and was compared with the 8 cm × 8 cm set using the same plan, evaluating the impact of catheters with no active dwell point (Figure 1). The applicator was cut into relevant squares with regular sizes except for set E. The catheter lengths were measured by the source ruler. The phantom with FF applicator set A was scanned using a Brilliance Big Bore CT scanner (Philips, Amsterdam, Netherlands) with 120 kV, 375 mA, 600FOV, and 1 mm slice width. The image resolution was 512 × 512 pixels.

### 2.2 Treatment planning

Four Treatment plans were generated on Oncentra Brachy (version 4.5.3, Nucletron B V)^21^ TPS with TG‐43 algorithm based on the same CT series as the algorithm did not consider CT values of the materials around sources: (A) two catheters with 2 cm active dwell length; (B) four catheters with 4 cm active dwell length; (C) six catheters with 6 cm active dwell length; (D) eight catheters with 8 cm active dwell length. All dwell position step size was 2.5 mm. The plans were prescribed with 5 Gy to cover the targets to avoid overdose at the skin surface and normalized at 0.5 cm point under MatriXX surface located at the center of the applicators. Dwell times were optimized with distance. Dose distributions were calculated using the TG‐43 algorithm. Planar doses at 0.36, 0.56, 1.36, and 2.36 cm under the MatriXX surface were exported for comparison, as the effective measuring plane of MatriXX was 0.36 cm under the surface.

### 2.3 ^192^Ir irradiation and measurement

**FIGURE 2:**
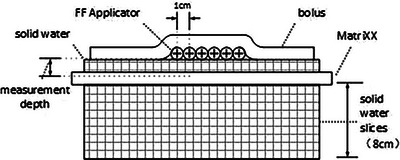
The experimental set of the phantom. Bolus with 0, 0.3, 0.5, 0.8, 1, 1.5 2, and 3 cm thickness were set upon the applicator with a 20 cm × 20 cm size. Solid water slices with 0, 0.2, 1, and 2 cm were set between the MatriXX surface and the applicator to get planar dose at 0.36, 0.56, 1.36, and 2.36 cm under MatriXX surface. 8 cm solid water slices were set under the MatriXX to offer full back scatter.

The MatriXX Evolution was calibrated using a 6MV x‐rays beam on a Varian Edge machine (Varian Medical Systems, Palo Alto, CA) as the dose dependence has been analyzed by other authors, which showed k_user_ difference between 4MV and 300kVp x‐rays was under 3%.^20^ The control unit part was covered with Pb protective aprons to avoid radiation damage to the board. The source activity was also controlled at a rather low level. The MicroSelectron HDR‐Brachy Unit with Iridium‐192 source was used in this study. The unit was calibrated using the well‐type ionization chamber. Dwell positions were verified before the experiment.^22^ Each plan was delivered with different solid water slices (0, 0.2, 1, and 2 cm) between the MatriXX surface and the applicators and bolus of different thickness (0, 0.3, 0.5, 0.8, 1, 1.5, 2, and 3 cm) as shown in Figure 2. The measurement depth was composed of the thickness of solid water and the effective measurement depth of MatriXX (0.36 cm). Therefore, the corresponding distance from source to measurement plane was 0.86, 1.06, 1.86, and 2.86 cm. A total of 128 plans (4 phantom sets × 4 planes × 8 bolus) were delivered for phantom sets A‐D. Phantom set E with no solid water slice and no bolus was irradiated using plan D for comparison. The applicator was placed away from the control unit region to reduce radiation dose to the unit and fixed by medical adhesive tape.

### 2.4 Dose distribution comparison

The planar dose and measured dose were compared in the software MyQA Platform (IBA Dosimetry, Schwarzenbruck, DE). The gamma index method evaluates the agreement between the calculated and measured dose distributions by utilizing the percent dose difference and distance to the agreement. It is a useful parameter for planar dose comparison. The planar dose exported from Oncentra was a 3D DICOM‐RT file, which was recognized as two DICOM‐RT files in MyQA. The first file was selected for comparison as it was the same as that in TPS. The measurement image solution was resampled from 7.6 to 1 mm to improve the image quality and data points.

## 3 RESULTS

### 3.1 Comparison between the full size and regular applicators

As shown in Table 1, the max dose, average dose and γ passing rate of phantom set D were higher than the corresponding results of irradiating the same phantom set using the same plan but with no solid water slice below and no bolus above the applicator. The max dose and average dose of phantom E were much closer to value of the plan in the TPS. As there were empty catchers with no dwell position in phantom E, which offering side scatter conditions. It is worth noting that there was no empty catcher around in most clinical case. So, the applicator used for QA measurement should be cut into a regular size in accordance with the applicator used in the treatment.

**TABLE 1:** The different dose index between phantom set D (regular size) and E (full size).

	Max dose (cGy)	Mean dose (cGy)	γ passing rate (3 mm/3%)
Plan	522.6	156.35	
Set D	455.8	138.8	82.1%
Set E	463.5	140.2	84.3%

### 3.2 Dose distribution comparison

The resolutions of DICOM dose images are of great importance when evaluating the mean dose and γ passing rate. Both regions of interest and resolution were enhanced during the analyzing process. The lengths of the square regions were mainly determined by the 10% isometric dose curve in TPS and with a slight enlargement (Table 2).

**TABLE 2:** The length of square regions of interest for different catchers and slices (cm).

Depth	Two catchers	Four catchers	Six catchers	Eight catchers
0.36	8.5	12	15	18
0.56	8.5	12	15	18
1.36	7.5	11	14.5	18
2.36	6	10	13.5	17

**FIGURE 3:**
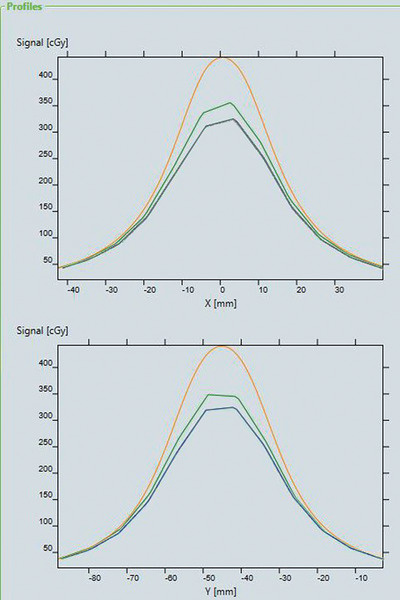
The cross lines at x and y direction for phantom A (two catchers case) with different thickness bolus at 0.56 depth. The orange line was the profile of the plan. The green and blue lines were the profile of the measurement with and without 3 cm bolus above the applicator.

The comparisons were conducted on the myQA Platform. The cross lines in the x and y directions were analyzed on image align window (Figure 3).

**FIGURE 4:**
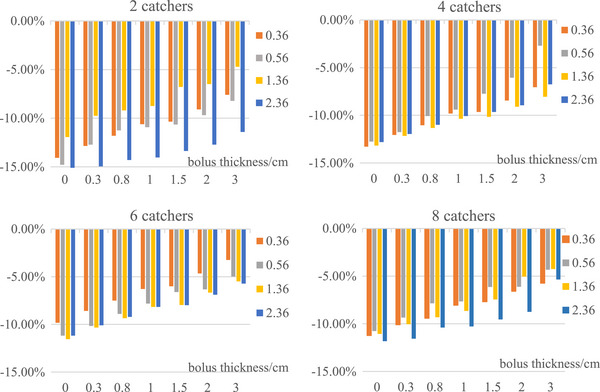
The average dose differences of four phantom sets at different depth with different bolus. (a) Two catchers (phantom A); (b) four catchers (phantom B); (c) six catchers (phantom C); (d) eight catchers (phantom D).

As shown in Figure 4, the measured mean doses were lower than the corresponding values calculated in TPS at all depths. The differences increased with decreasing field size, which indicated that the dose disagreement would be more significant in small target cases. However, the differences became smaller after adding the bolus. As the bolus thickness increased, the differences decreased, indicating that lack of backscattering in the irradiations contributed to the dose differences between the measurements and the corresponding TG‐43 based dose calculations, thus adding the backscattering materials improved the agreements. Our measurement results showed that to achieve the agreement within 10% at the depth of 0.56 cm, which was close to the prescription normalization point, 2, 1, 0.8, and 0.3 cm bolus should be added for phantom A, B, C and D, respectively. No correlation was found between the average dose difference and the depth.

Gamma analysis is widely used in radiotherapy dose verification. The statistical values of Gamma analysis with 3 mm/3% criterion were summarized in Table 3 for the conducted measurements. The gamma value(γ) increased as the bolus thickness increased and the depth decreased. As the depth increased, the dose level decreased, resulting in a low passing rate. The γ passing rate of relevant dose would be much higher (more than 95%) if max dose of measurement was normalized to the same value with the max dose exported from TPS. 3 mm/3% was applied in this study according to previous research, although 2 mm/2% was commonly applied in stereotactic body radiation therapy, which has the similar dose pattern with much higher dose per fraction in target and rapid falloff outside the field. γ passing rate with 2 mm/2% was also evaluated in this paper. The gamma value of the absolute dose was much lower than 90%.

**TABLE 3:** Summary of the absolute dose γ passing rate in 3 mm/3%.

3%/3 mm			Bolus (cm)						
Source length		Measured depth (cm)	0	0.3	0.8	1	1.5	2	3.0
**Two catchers**	2 cm	0.36	94.9	95.1	95.4	95.6	95.9	96.2	96.8
		0.56	93.0	94.1	94.8	96.2	96.6	97.6	99.6
		1.36	73.5	79.6	80.4	79.2	84.4	85.4	86.7
		2.36	0.0	4.6	12.4	15.5	23.2	30.9	46.4
**Four catchers**	4 cm	0.36	86.5	87.2	87.8	88.5	88.7	89.4	90.5
		0.56	89.0	89.5	90.4	90.7	91.6	92.4	94.1
		1.36	76.8	79.4	81.0	83.7	83.3	85.7	87.1
		2.36	31.5	42.0	52.1	64.9	66.2	71.9	76.5
**Six catchers**	6 cm	0.36	87.1	87.6	88.2	88.6	89.1	89.7	91.0
		0.56	85.4	85.2	87.5	89.3	90.2	92.2	92.7
		1.36	76.0	78.5	80.5	82.9	83.3	86.1	88.2
		2.36	54.4	61.9	66.1	74.5	72.0	77.6	81.5
**Eight**	8 cm	0.36	82.1	82.4	83.0	83.2	83.8	84.4	85.5
		0.56	78.0	83.5	84.4	84.7	85.6	86.4	90.5
		1.36	69.5	83.0	83.9	84.3	86.2	87.1	89.7
		2.36	59.6	68.5	70.7	72.2	73.8	79.7	88.3

## 4 DISCUSSION

This paper measured planar dose of FF applicator for different target sizes in keloid HDR‐brachytherapy with MatriXX device. The results in Figures 3 and 4 show the dose deficiencies in superficial cases resulting from the insufficient scatter environment. The TG‐43 formulism assumes full phantom scattering environment around radioactive source and assumes water as the phantom material, leading to overestimated doses for cases lack of full phantom scattering, such as HDR keloid treatment using Freiburg flap without adequate bolus on top of the skin surface. Additionally, the TG‐43 algorithm‐based dose calculation does not take into account the differences from water of the FF applicator material composition, the presence of air gap between the spheres, the source cable material details, material compositions of the MatriXX device and solid water material, resulting in additional calculation differences in TPS. Similar results were also derived in some previous studies, and Table 4 summarizes their experimental setups and results.

**TABLE 4:** Experimental setup and results of previous studies.

	Radiation field/FF applicator size	Prescription/depth in tissue	Tools	Main results
**Vijande et al**.^12^	5 cm ×5 cm	5 mm	Penelope 2008 EBT2	<5% difference
**Raina et al**.^11^	4 cm ×4 cm, 7 cm ×7 cm, 12 cm ×12 cm	0.5 cm 1 cm 1.5 cm	Liquid ion chamber array (PTW)	8.5% (0.5 cm prescription depth) 12.5% (1.0 cm) 15% (1.5 cm)
**Aldelaijan et al**.^9^	4 cm ×4 cm, 11 cm ×11 cm Full size for measurement	6 Gy/1 cm	EBT3	<3% after adding 2 cm bolus for small target and 1 cm for large target
**Yewondwossen** ^13^	(1) 3 channels (2) 10 cm ×10 cm box (3) C shape Full size for measurement	2 Gy/2 cm	MatriXX	<3.7% average dose difference >95% γ passing rate with 3 mm/3% criteria
**Ohta et al**.^10^	5 cm ×5 cm Larger size for measurement	6 Gy/2 mm	RPL‐glass dosimeter PHITS	Calculated dose was underestimated by 13.3 to 21.8%

This paper compared the dosimetric distributions between full size and regular cut FF applicators, showing that regular size cutting was necessary as the applicator material would affect dose distribution, which might be a reason for differences with previous research in this paper. More attention should be paid to the applicator size in future work as most studies used full size applicator for measurement. Another reason for the differences might be the x‐ray energy used for MatriXX calibration. The characterization and use of MatriXX for brachytherapy dosimetric quality assurance was investigated by Yewondwossen. In that study, little energy dependence was detected as the maximum variation of calibration factor k_user_ between 300kVp and 4MV was <3%. A 300kVp beam was applied for calibration in their study,^13^ while 6MV was applied in this study.^23^ The use of a 6 MV photon beam for the calibration was investigated by Huang et al.^24^ and was found to be feasible and reliable.

Likhacheva et al. surveyed the skin surface brachytherapy practice patterns, and found that the prescription ranged from 2 to 10 Gy per fraction with depth ranged from 1 mm to 1 cm.^25^ 5 Gy per fraction at 0.5 cm was clinically applied in our institution. Bolus was applied instead of solid water slice to simulate treatment situation. Bolus of more than 3 cm thickness was not applied in our study as it was not commonly used in clinics, although more bolus would offer full scatter condition.

## 5 CONCLUSION

This paper proposes a verification method using MatriXX for end‐ to‐end dosimetric evaluation to determine the minimal amount of bolus to be used to achieve less than 10% discrepancy between treatment and TG‐43 based dose calculation. It is found that for different target size, bolus of different thickness is needed to improve the actual dose coverage for plans generated with the TG‐43 algorithm, and the proposed measurement method may provide the needed information. This study provides only physical results, while biological experiments should be done before clinical use. More studies are needed to draw more definitive clinical recommendations on the amount of bolus for different target size.

## AUTHOR CONTRIBUTIONS

The author confirm contribution to the paper as follows: Jie Ni has made substantial contributions to the design and draft of the work. Guanghui Gan has made contributions to the data acquisition and analysis. Xiaoting Xu has revised the paper. All authors reviewed the results and finally approved the manuscript.

## CONFLICT OF INTEREST STATEMENT

All authors declare that there are no conflicts of interests
